# Monetary Matched Incentives to Encourage the Purchase of Fresh Fruits and Vegetables at Farmers Markets in Underserved Communities

**DOI:** 10.5888/pcd10.130124

**Published:** 2013-11-14

**Authors:** Suzanne Lindsay, Jennifer Lambert, Tanya Penn, Susan Hedges, Kristine Ortwine, Anchi Mei, Tracy Delaney, Wilma J. Wooten

**Affiliations:** Author Affiliations: Jennifer Lambert, Tanya Penn, Susan Hedges, Kristine Ortwine, Institute for Public Health, San Diego State University, San Diego, California; Anchi Mei, International Rescue Committee, San Diego, California; Tracy Delaney, Wilma J. Wooten, Health and Human Services Agency, County of San Diego, California. Ms Delaney is now affiliated with the Public Health Institute, Oakland, California.

## Abstract

**Introduction:**

Farmers market programs may increase access to more healthful foods and reduce the high prevalence of obesity in low-income communities. The objective of this study was to examine outcomes of the Fresh Fund farmers market program serving low-income neighborhoods in San Diego, California.

**Methods:**

Through its Farmers Market Fresh Fund Incentive Program, the County of San Diego Health and Human Services Agency offered monetary incentives to government nutrition assistance recipients to purchase fresh produce at 5 farmers markets. Participants enrolled at participating markets from June 1, 2010, through December 31, 2011; they completed baseline and follow-up surveys of daily consumption and weekly spending on fruits and vegetables. We examined enrollment, participation, participant health perceptions, and vendor revenue.

**Results:**

During the study period, 7,298 eligible participants enrolled in Fresh Fund; most (82%) had previously never been to a farmers market. Among 252 participants with matched surveys at baseline and 12-month follow-up, the proportion who reported their diet to be “healthy” or “very healthy” increased from 4% to 63% (*P* < .001); nearly all (93%) stated that Fresh Fund was “important” or “very important” in their decision to shop at the farmers market. Vendors reported that 48% of all market revenue they received was received through the Fresh Fund program. At 2 markets, revenue from June 1, 2011, through January 31, 2012, increased by 74% and 68% compared with revenue from June 1, 2010, through January 31, 2011.

**Conclusion:**

Participants in the Fresh Fund program self-reported increases in daily consumption and weekly spending on fruits and vegetables, and vendors at participating farmers markets also increased their revenue.

## Introduction

In recent decades, the prevalence of overweight and obesity has increased in the United States (1,2). The National Health and Nutrition Examination Survey shows that in 2009–2010, more than 78 million adults and roughly 12.5 million children and adolescents were obese (3); these Americans are at greater risk for adverse health outcomes such as type 2 diabetes and coronary heart disease and overall morbidity and mortality (4–6). Consuming adequate amounts of fruits and vegetables is an essential part of reducing poor health outcomes (7,8); however, in 2009 only 36% of adults consumed the recommended 2 or more servings of fresh fruit per day, and only 26% consumed 3 or more servings of vegetables per day (9). Low-income Americans are particularly at risk for poor dietary habits and related health conditions because of lack of access to affordable produce (10–13). 

In 2008, the City Heights Farmers Market Fresh Fund (Fresh Fund) program was established in a low-income refugee community in San Diego County by the San Diego International Rescue Committee. It was the first program in San Diego to encourage the purchase of fresh fruits and vegetables at a farmers market by eligible participants using government nutrition assistance programs. In April 2010, the Fresh Fund program was expanded to 1 additional market in north San Diego. Later that year, San Diego County was awarded funding through the Centers for Disease Control and Prevention’s (CDC’s) 2-year initiative, Communities Putting Prevention to Work (CPPW), to help reduce obesity and prevent chronic disease (14). One of San Diego’s CPPW-funded interventions was an enhancement of the 2 existing Fresh Fund programs and expansion to 3 additional markets in low-income neighborhoods, 2 of which also have large immigrant and refugee populations. The objective of this study was to examine patterns of enrollment and market visits, participants’ self-reported dietary changes while participating in the program, and the economic benefits of the program, particularly for the farmer vendors.

## Methods

This study was a practice-based evaluation conducted in partnership with community-based practitioners for the purpose of understanding how interventions can be incorporated and sustained in existing community practice settings. We used mixed methods and repeated measures and collected data from Fresh Fund participants who enrolled from June 1, 2010, through December 31, 2011. The study period included an extra month (January 2012) to capture data on Fresh fund visits and money spent by participants who enrolled in December 2011 but continued to purchase produce in January 2012. With CPPW support, the Fresh Fund expansion was designed by the County of San Diego Health and Human Services Agency (HHSA), the Division of Child Development and Community Health at the University of California San Diego, and the International Rescue Committee. Five markets operated during the study period (June 1, 2010, through January 31, 2012), 2 of which were operational at the beginning of the study period. The goal of the Fresh Fund expansion program was to enroll 3,000 eligible participants. HHSA gave approval for the Institute for Public Health at San Diego State University (SDSU) to analyze Fresh Fund program data for evaluation purposes. The SDSU institutional review board reviewed and approved the evaluation plan.

### Eligibility, enrollment, and participation data

Government assistance recipients in the Supplemental Nutrition Assistance Program (SNAP), the Special Supplemental Nutrition Program for Women, Infants, and Children (WIC), and Supplemental Security Income (SSI) were eligible to participate in Fresh Fund, which allowed them to use their SNAP, WIC, or SSI benefits at 5 farmers markets. Eligible participants enrolled in the program at a Fresh Fund booth at the market where they were able to use SNAP or SSI through electronic benefit transfer (EBT) cards, WIC vouchers, cash, or debit or credit cards to buy Fresh Fund “purchased” tokens. They were then given differently marked “incentive” tokens (up to $20 per month) to match the amount of their purchased tokens. Only eligible government assistance recipients could purchase tokens and receive incentive tokens (including tokens purchased with cash). The Fresh Fund tokens could be spent only at certain types of vendors: fresh produce vendors (farmers) and vendors that provided certain types of healthful packaged food (eg, eggs, bread, meat). Participants did not have to spend all of their purchased and incentive tokens on the same day they were purchased (ie, tokens could be saved for use at future markets). Among all 5 markets, farmers represented 19% of all vendors; nonfarmer vendors included vendors of arts and crafts, hot foods, and packaged foods. Eligibility for incentives was verified and tracked by using an online database in which Fresh Fund personnel recorded the amount of money used by each participant to purchase tokens each week and their receipt of matched incentive tokens. Vendors accepted these tokens from participants and exchanged them with market management at the end of the day for reimbursement. Enrollment data collected at the Fresh Fund booth each week were used to evaluate participant enrollment and market use. Participant enrollment was concurrent with outreach and media efforts that began in June 2011, including 22 weeks of television advertisements, direct-mail flyers sent 6 times to 130,000 homes in neighborhoods adjacent to the markets, and posters on buses and at bus stops. The media campaign described the value of eating fresh fruits and vegetables and the components of the Fresh Fund program (www.HealthyWorks.org). Fresh Fund program staff at each market met with local nonprofit community-based organization (CBO) managers to describe the program and encourage them to promote it. They also provided Fresh Fund informational flyers to CBOs for distribution to their clients.

### Self-reported data on participants at baseline and follow-up

Participants visiting the Fresh Fund enrollment booth were asked to complete a voluntary survey (Appendix) during their first enrollment visit (baseline) and at approximately 3-month intervals for as long as they participated in the Fresh Fund program. At every visit to the market, eligible participants were required to check in at the Fresh Fund booth, where the date of each visit and the details of monetary exchanges were recorded. The baseline and follow-up surveys used numeric scales and were available in 5 languages (English, Spanish, Vietnamese, Somali, and Chinese). The surveys were conducted via paper and pencil or if necessary, because of literacy issues, through an interview. Surveys were coded by Fresh Fund program staff according to a unique subject identification, which allowed comparisons over time. Follow-up surveys were grouped into 2 categories: 3-to-6 month follow-up surveys (completed between 3 and 6.5 months after enrollment) and a 12-month-or-more follow-up survey (completed 11.5 months after enrollment or later). If participants completed multiple follow-up surveys in 1 or both periods, we used the most recent survey data. Among the 1,697 participants who visited the market multiple times for at least 3 months, 908 completed both a baseline and 3-to-6–month follow-up survey (54% response rate). Among the 582 participants who visited the market multiple times for 12 months or more, 252 completed both a baseline and follow-up survey (43% response rate). The reported demographics (ie, sex, age, race/ethnicity, number of people in household) of those who submitted follow-up surveys were similar to those who submitted baseline surveys. Baseline and follow-up data were compared for 3 survey questions: 1) How much on average do you spend on fresh fruits and vegetables per week?, 2) On average, how many servings of fruits and/or vegetables do you usually eat each day?, and 3) In general, how healthy would you say your overall diet is?

### Data on vendor revenues

Data were collected from 448 vendors at the 5 participating markets. As part of their agreement to sell at the market, each vendor was required by the market management to submit a report at the end of each market day. These vendor reports documented that the business was present at the market and the amount of money each vendor received during the market day from both Fresh Fund and non-Fresh Fund purchases. The amount of money received through the Fresh Fund program was calculated by counting both purchased and incentive tokens submitted by vendors for reimbursement.

### Data management and statistical analysis

Enrollment data, baseline and follow-up survey data, and vendor data were entered into databases by Fresh Fund program staff and delivered de-identified to the evaluation team at the Institute for Public Health. We calculated descriptive statistics for all participant data, including enrollment, demographics, number of visits, and types and amounts of money exchanged and used to purchase products from vendors by market and by month. We used χ^2^ tests to compare baseline and follow-up survey responses. Because the data on vendor revenue were not normally distributed, we used the Wilcoxon signed-rank test to compare these data across markets and over time. An α level of .05 was applied for all analyses. Data were analyzed using SPSS version 19 (International Business Machine Corp, Armonk, New York).

## Results

A total of 7,298 eligible participants (Table 1) enrolled in Fresh Fund, exceeding the program goal of 3,000 by 143%. Most participants (82%) had previously never been to a farmers market. Enrollment increased substantially during summer 2011, and the largest enrollment was in August 2011 (n = 1,089). Participants averaged 2.8 visits, markets averaged 72 Fresh Fund participants per day, and 21,025 monetary exchanges took place during the study period. The highest number of visits (n = 2,145) occurred in August 2011 (Figure). Almost half (46%) of participants returned to the market more than once; 17% visited 5 or more times. The 2 markets operating during the entire study period had a 95% increase in enrollment and a 139% increase in visits in August 2011 compared with August 2010. More than one-third of monetary exchanges (39%) were cash exchanges, 32% were EBT exchanges, and 26% were WIC voucher exchanges. Through these exchanges, the Fresh Fund program provided $680,873 in total market spending power ($350,512 in purchased tokens plus $330,361 in incentive tokens) to eligible government nutrition assistance participants at the 5 markets, for an average of $34 per participant per visit, and an average of $93 per participant throughout the study.

**Table 1:** Characteristics of San Diego County Fresh Fund Participants, June 1 2010, Through December 31, 2011 (N = 7,298)

Characteristic	No. (%)
**Sex (n = 7,285)^a^**	
Male	1,121 (15.4)
Female	6,164 (84.6)
**Race/ethnicity (n = 7,298)**	
African American	485 (6.6)
American Indian or Alaska Native	13 (0.2)
Asian	754 (10.3)
Vietnamese	787 (10.8)
Pacific Islander/Native Hawaiian	46 (0.6)
East African	212 (2.9)
Multiracial	73 (1.0)
White	1,316 (18.0)
Hispanic or Latino	3,612 (49.5)
**No. of people in household (n = 7,293)^a^**	
1 or 2	1,625 (22.3)
3 or 4	3,119 (42.8)
5 or 6	2,025 (27.8)
≥7	524 (7.2)
**Participant type/eligibility (n = 7,298)**	
SNAP/CalFresh	1,958 (26.8)
SSI	1,248 (17.1)
WIC	4,092 (56.1)
**Participant age, y (n = 7,275)^a^**	
<24	984 (13.5)
25–34	2,826 (38.8)
35–44	1,546 (21.3)
45–54	542 (7.5)
55–64	448 (6.2)
≥65	929 (12.8)
**Participant residence by HHSA region (n = 7,137)** a	
Central	3,498 (49.0)
East	274 (3.8)
North Central	1,122 (15.7)
North Coastal	1,563 (21.9)
North Inland	481 (6.7)
South	199 (2.8)

Abbreviations: SNAP, Supplemental Nutrition Assistance Program; SSI, Supplemental Security Income; WIC, Special Supplemental Nutrition Program for Women, Infants, and Children; HHSA, Health and Human Service Agency.

a Does not total 7,298 because of unanswered questions or missing data.

**Figure:**
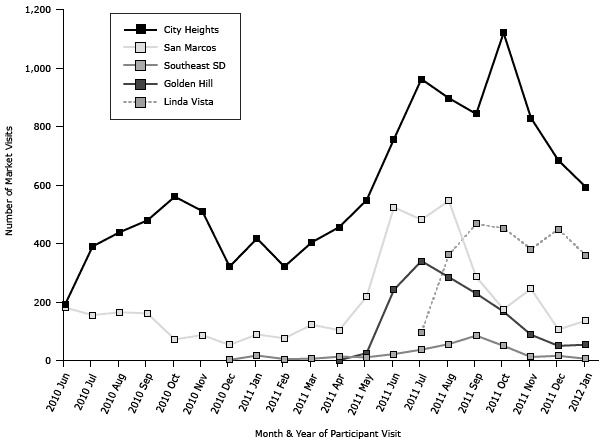
Number of Fresh Fund market visits by market location and month (20,089 total visits), June 1, 2010, through January 31, 2012. The Southeast San Diego Market opened in December 2010, the Golden Hill Market opened in April 2011, and the Linda Vista Market opened in July 2011. A media marketing campaign was initiated in June 2011. **Table:** 

Month and Year	City Heights	San Marcos	Southeast SD	Golden Hill	Linda Vista	Total
2010 Jun	192	181	—	—	—	373
2010 Jul	390	155	—	—	—	545
2010 Aug	439	165	—	—	—	604
2010 Sep	479	161	—	—	—	640
2010 Oct	560	72	—	—	—	632
2010 Nov	511	87	—	—	—	598
2010 Dec	321	53	2	—	—	376
2011 Jan	417	89	17	—	—	523
2011 Feb	321	76	5	—	—	402
2011 Mar	404	122	6	—	—	532
2011 Apr	456	104	13	1	—	574
2011 May	548	218	11	25	—	802
2011 Jun	757	524	22	242	—	1,545
2011 Jul	962	481	37	340	95	1,915
2011 Aug	897	546	56	285	361	2,145
2011 Sep	843	286	85	229	467	1,910
2011 Oct	1,120	175	50	167	452	1,964
2011 Nov	829	245	12	88	381	1,555
2011 Dec	684	106	16	50	449	1,305
2012 Jan	593	136	6	54	360	1,149

Abbreviation: —, market not open.

### Comparison of baseline and follow-up surveys

The distribution of survey responses on daily fruit and vegetable consumption changed significantly between baseline and follow-up. The percentage of respondents who reported eating 5 or more daily servings of fruits and vegetables increased from 23.7% to 29.6% for 3-to-6–month participants (Table 2) and 19.4% to 24.2% for 12-month participants (Table 3). The distribution of responses on self-reported perceptions of diet also changed significantly for both groups of participants. The percentage of respondents who reported “healthy or very healthy diets” increased from 33.3% to 68.6% for 3-to-6–month participants and from 4.0% to 63.1% for 12-month participants.

**Table 2:** San Diego County Fresh Fund Participant Matched Baseline and 3-to-6–Month Follow-up Survey Data, June 1, 2010, Through January 31, 2012

Survey Question	Matched Baseline, No. (%)	Matched 3–6 Month Follow-up, No. (%)	*P* Value^a^
**How much on average do you spend on fresh fruits and vegetables per week?**			
<$20	255 (28.1)	237 (26.1)	<.001
$20-$29	283 (31.2)	287 (31.6)	
≥$30	370 (40.7)	384 (42.3)	
**On average, how many servings of fruits and/or vegetables do you usually eat each day?**			
0–2	258 (28.4)	214 (23.6)	<.001
3 or 4	434 (47.8)	426 (46.9)	
≥5	216 (23.8)	268 (29.5)	
**In general, how healthy would you say your overall diet is?**			
Very unhealthy/unhealthy	339 (37.3)	20 (2.2)	<.001
Average	273 (30.1)	271 (29.8)	
Healthy/very healthy	296 (32.6)	617 (68.0)	

a χ^2^ test was used to test for significance; an α level of .05 was applied for all analyses.

**Table 3:** San Diego County Fresh Fund Participant Matched Baseline and 12-Month Follow-up Survey Data, June 1, 2010, Through January 31, 2012

Survey Question	Matched Baseline, No. (%) (n = 252)	Matched 12-Month Follow-up, No. (%) (n = 252)	*P* Value^a^
**How much on average do you spend on fresh fruits and vegetables per week?**			
<$20	91 (36.1)	53 (21.0)	<.001
$20-$29	79 (31.3)	79 (31.3)	
≥$30	82 (32.5)	120 (47.6)	
**On average, how many servings of fruits and/or vegetables do you usually eat each day?**			
0-2	82 (32.5)	61 (24.2)	<.001
3 or 4	121 (48.0)	130 (51.6)	
≥5	49 (19.4)	61 (24.2)	
**In general, how healthy would you say your overall diet is?**			
Very unhealthy/unhealthy	170 (67.5)	13 (5.2)	<.001
Average	72 (28.6)	80 (31.7)	
Healthy/very healthy	10 (4.0)	159 (63.1)	

a χ^2^ test was used to test for significance; an α level of .05 was applied for all analyses.

Nearly all participants (93%) stated that Fresh Fund was either “important” or “very important.” Most of participants at 3 to 6 months (71%) said that they would be “somewhat” or “completely likely” to shop at the farmers market without the Fresh Fund incentive, whereas about half (55%) of the participants at 12 months stated they would be “somewhat” or “completely likely” to continue.

### Vendor revenue

Vendors reported $1.7 million in sales at the 5 markets during the study period, an average of $6,133 per market day (including both Fresh Fund and non-Fresh Fund sales). Fresh Fund tokens purchased with government assistance funding represented 14% of the revenue, incentive tokens represented 22% of revenue, and tokens purchased with personal cash or credit by Fresh Fund–eligible participants represented 12% of revenue. Thus, 48% of all market revenue received by vendors was received by encouraging government nutrition assistance–eligible persons to shop at the markets through the Fresh Fund program. The remaining 52% of the revenue was from non-Fresh Fund shoppers, or from Fresh Fund shoppers not using the Fresh Fund token-purchasing system. For the 18 farmer vendors who were present and selling at the markets from June 1, 2010, through January 31, 2011, and from June 1, 2011, through January 31, 2012, the average revenue per market day increased from $418.88 in the first period to $566.84 in the second period (*P* = .006). Although nonfarmer products could not be purchased with Fresh Fund tokens, average nonfarmer revenue also increased during the study period from $107.86 to $150.29 per market day (n = 33 nonfarmer vendors, *P* < .001). Although farmers comprised only 19% of the total number of vendors at the markets, their revenue during the study period accounted for 62% of the total revenue for all vendors.

## Discussion

This practice-based evaluation demonstrated that the combination of a $20 matched incentive, media marketing efforts, and collaboration with local community-based organizations was successful in bringing 7,298 low-income government assistance recipients to 1 of 5 markets, most of them for the first time. Vendors at the markets also benefited, with more than $1.7 million in sales, almost half of which (approximately $800,000) was provided by Fresh Fund participants. Two long-standing farmers markets open throughout the study period provided an opportunity for comparison of revenue generation over time. Market 1 had a 74% increase in revenue in the period June 1, 2011, through January 31, 2012, compared with June 1, 2010, through January 31, 2011, while Market 2 had a 68% increase in revenue in the same period. Eligible participants used personal cash for 12% of their purchases, indicating that the added value of the Fresh Fund program went beyond simply infusing government assistance money into the market. Participants reported increases in weekly spending for fruits and vegetables, increases in daily consumption of fruits and vegetables, and better overall dietary health, a finding that has been documented in similar studies (15). Interestingly, participants who continued in Fresh Fund for at least 12 months were demographically similar to those with baseline and 3-to-6–month follow-up surveys, yet they reported a significantly poorer baseline perception of overall dietary health; only 4% reported their diet to be “healthy” or “very healthy” at baseline. This finding may imply that those who used the Fresh Fund program longest were more likely to need it the most, and it warrants further investigation. Finally, in terms of sustainability, all 5 markets continue to encourage the use of government nutritional assistance programs for the purchase of fresh fruits and vegetables, although only 1 market continues to offer a modest incentive provided by a local nonprofit whose mission is to increase access to nutritious food. Previous research has demonstrated that low-income community members can be encouraged to shop at local farmers markets through the promotion of government nutritional assistance programs (SNAP, WIC, SSI) to purchase fresh fruits and vegetables (16,17). This evaluation adds to the current knowledge by providing information on enrollment trends, visit patterns, self-reported improvement in diet, and vendor benefits.

Implementation of the Fresh Fund program also created challenges. Some markets reported long lines and some difficulty among shoppers in understanding the token system. Because of the way data were collected, a Fresh Fund eligible participant could visit a market and spend his or her own money but not register at the Fresh Fund booth and therefore not be counted as a return visitor. Thus, the follow-up data may undercount the number of repeat visits. During Fresh Fund vendor interviews, some vendors suggested the use of an electronic management system rather than the manual token system. Use of new technologies such as swipe cards has been shown to increase sales for SNAP participants in several local farmers markets in one Arizona region, highlighting the benefits that can be gained not only by consumers but by vendors as well (18).

Incentive programs that market and encourage the purchase of local fresh fruits and vegetables by low-income populations are relatively new, and more research and evaluation are needed. Further analysis is needed to determine characteristics of farmers markets or program interventions that might encourage or discourage participation. For example, 1 market in our study may have been successful because the WIC office in that neighborhood was active in educating clients about the program. Another market may have been successful because it sold ethnic food products familiar to community members (including refugees). Studies examining whether an incentive of less than $20 a month would be sufficient for positive participant outcomes would also be valuable. Incentivizing low-income government assistance–eligible participants to purchase fresh fruits and vegetables at local farmers markets has the ultimate potential to reduce childhood and adult obesity and the long-term chronic disease burden in this population. Further research and evaluation is needed to determine whether encouraging the purchase of fresh fruits and vegetables leads to actual changes in consumption and diet, and in turn, reductions in obesity and improved health.

## Appendix. Enrollment and Follow-up Survey Questions, Study on Farmers Market Fresh Fund Incentive Program, San Diego, California, June 1, 2010, Through January 31, 2012

**Table:**

Enrollment Survey Questions	Follow-Up Survey Questions
**1) How often do you come to this farmers market?**	**1) How often do you come to this farmers market?**
a. This is my first time at this market.	a. This is my first time at this market since I enrolled in the Fresh Fund.
b. Every week	b. Every week
c. Once a month	c. Once a month
d. Twice a month	d. Twice a month
e. 1–2 times a season	e. 1–2 times a season
f. Missing/no answer/refused/don’t know	f. Missing/no answer/refused/don’t know
**2) How did you hear about Fresh Fund?**	**2) How important is the Fresh Fund in your decision to come to this market?**
a. Word of mouth	a. Very important (I wouldn’t have come without it)
b. WIC	b. Important
c. FRC	c. Somewhat important
d. Media	d. Not important (The Fresh Fund did not affect my decision to come to this market. I would have come without it.)
e. Flyers	e. Other _____
f. Other _____	f. Missing/no answer/refused/don’t know
g. Missing/no answer/refused/don’t know	—
**3) How likely are you to shop at this Farmers Market if you can still use EBT/WIC, but without the Fresh Fund incentive?**	**3) How likely are you to shop at this Farmers Market if you can still use EBT/WIC, but without the Fresh Fund incentive?**
a. Completely likely	a. Completely likely
b. Somewhat likely	b. Somewhat likely
c. Somewhat unlikely	c. Somewhat unlikely
d. Completely unlikely	d. Completely unlikely
e. Missing/no answer/refused/don’t know	e. Missing/no answer/refused/don’t know
**4) How much on average do you spend on fresh fruits and vegetables per week?**	**4) How much on average do you spend on fresh fruits and vegetables per week?**
a. Less than $10	a. Less than $10
b. $10–$19	b. $10–$19
c. $20–$29	c. $20–$29
d. $30–$39	d. $30–$39
e. $40 or more	e. $40 or more
f. Missing/no answer/refused/don’t know	f. Missing/no answer/refused/don’t know
**5) On average, how many servings of fruits and/or vegetables do you usually eat each day? (1 serving = 1 cup raw, leafy vegetables or ½ cup raw or cooked other fruits/vegetables, or 1 piece)**	**5) On average, how many servings of fruits and/or vegetables do you usually eat each day? (1 serving = 1 cup raw, leafy vegetables or ½ cup raw or cooked other fruits/vegetables, or 1 piece)**
a. Less than 1 serving a day	a. Less than 1 serving a day
b. 1–2 servings a day	b. 1–2 servings a day
c. 3–4 servings a day	c. 3–4 servings a day
d. 5+ servings a day	d. 5+ servings a day
e. Missing/no answer/refused/don’t know	e. Missing/no answer/refused/don’t know
**6) Of all fruits and vegetables you buy each week, how much comes from a Farmers Market?**	**6) Of all fruits and vegetables you buy each week, how much comes from a Farmers Market?**
a. Less than 25%	a. Less than 25%
b. 25%–49%	b. 25%–49%
c. 50%–75%	c. 50%–75%
d. 75% or more	d. 75% or more
e. Missing/no answer/refused/don’t know	e. Missing/no answer/refused/don’t know
**7) In general, how healthy would you say your overall diet is?**	**7) In general, how healthy would you say your overall diet is?**
a. Very healthy	a. Very healthy
b. Healthy	b. Healthy
c. Average	c. Average
d. Unhealthy	d. Unhealthy
e. Very unhealthy	e. Very unhealthy
f. Missing/no answer/refused/don’t know	f. Missing/no answer/refused/don’t know
